# Correlative Fluorescence and Raman Microscopy to Define Mitotic Stages at the Single-Cell Level: Opportunities and Limitations in the AI Era

**DOI:** 10.3390/bios13020187

**Published:** 2023-01-26

**Authors:** Csaba Voros, David Bauer, Ede Migh, Istvan Grexa, Attila Gergely Végh, Balázs Szalontai, Gastone Castellani, Tivadar Danka, Saso Dzeroski, Krisztian Koos, Filippo Piccinini, Peter Horvath

**Affiliations:** 1 Synthetic and Systems Biology Unit, Biological Research Centre (BRC), Temesvári krt. 62, H-6726 Szeged, Hungary; 2 Institute of Biophysics, Biological Research Centre (BRC), Temesvári krt. 62, H-6726 Szeged, Hungary; 3 Department of Medical and Surgical Sciences (DIMEC), University of Bologna, Via G. Massarenti 9, I-40126 Bologna, Italy; 4 Department of Knowledge Technologies, Jozef Stefan Institute, Jamova Cesta 39, SI-1000 Ljubljana, Slovenia; 5 Scientific Directorate, IRCCS Istituto Romagnolo per lo Studio Dei Tumori (IRST) “Dino Amadori”, Via P. Maroncelli 40, I-47014 Meldola, Italy; 6 Institute for Molecular Medicine Finland-FIMM, Helsinki Institute of Life Science-HiLIFE, University of Helsinki, FI-00014 Helsinki, Finland; 7 Single-Cell Technologies Ltd., Temesvári krt. 62, H-6726 Szeged, Hungary

**Keywords:** microscopy, Raman spectroscopy, single-cell analysis, phenotypic discovery, mitosis, machine learning

## Abstract

Nowadays, morphology and molecular analyses at the single-cell level have a fundamental role in understanding biology better. These methods are utilized for cell phenotyping and in-depth studies of cellular processes, such as mitosis. Fluorescence microscopy and optical spectroscopy techniques, including Raman micro-spectroscopy, allow researchers to examine biological samples at the single-cell level in a non-destructive manner. Fluorescence microscopy can give detailed morphological information about the localization of stained molecules, while Raman microscopy can produce label-free images at the subcellular level; thus, it can reveal the spatial distribution of molecular fingerprints, even in live samples. Accordingly, the combination of correlative fluorescence and Raman microscopy (CFRM) offers a unique approach for studying cellular stages at the single-cell level. However, subcellular spectral maps are complex and challenging to interpret. Artificial intelligence (AI) may serve as a valuable solution to characterize the molecular backgrounds of phenotypes and biological processes by finding the characteristic patterns in spectral maps. The major contributions of the manuscript are: (*I*) it gives a comprehensive review of the literature focusing on AI techniques in Raman-based cellular phenotyping; (*II*) via the presentation of a case study, a new neural network-based approach is described, and the opportunities and limitations of AI, specifically deep learning, are discussed regarding the analysis of Raman spectroscopy data to classify mitotic cellular stages based on their spectral maps.

## 1. Introduction

Nowadays, most of the discoveries in medicine and biology rely on the examination of cells using super-resolution microscopes and other advanced techniques for single-cell analysis [1]. For instance, studying the effects of chemical compounds (*a*) on the cells of different phenotypes [2], and (*b*) on cells at different cellular stages [3], is the basis of new drug development in several areas, including oncology. Thus, the reliable detection of cellular phenotypes has become a fundamental step of the pipeline [4]. Regarding cellular processes, mitosis is considered as being crucial, especially in oncology where cancer cells are characterized by an uncontrolled proliferation rate [5].

According to Aristotle’s principle, “*The whole is greater than the sum of its parts*” [6]. However, in 2022, no single instrument is capable of analyzing all aspects of cells and tissues in detail. Thus, for a comprehensive characterization of a sample, a combination of advanced techniques and multi-modal data analysis approaches are often required. *Raman spectroscopy* is a label-free, non-invasive, and in vivo method ideal for single-cell assessment to obtain chemical information [7]. It is based on the inelastic light scattering phenomenon to detect an analyte via molecular bond vibrations, and it is one of the main marker-free analytical techniques utilized in optical metrology to provide insight into the structure and composition of tissues and cells at the molecular level. It produces Raman spectra that serve as a fingerprinting tool for a wide range of compounds [8]. *Raman microscopy* combines Raman spectroscopy with an optical microscope [9]. *Correlative microscopy* combines different light, electron, and/or other microscopy techniques for a more detailed examination of samples [10]. The individual microscopy techniques can be executed at different times, and eventually, additional biological preparations can be applied between the different examinations. The assessment yields different types of data that provide morphological, structural, functional, and biological information, and they can be used to establish a fingerprint of each single cell. Traditionally, the combination of light microscopy (LM) and/or fluorescence microscopy (FM) with electron microscopy (EM) is the most common form of correlative microscopy [11]. However, EM is a destructive technique. Therefore, as a current trend, correlative microscopy analyses increasingly combine LM/FM with non-invasive Raman micro-spectroscopy [12].

The first work combining FM (specifically, a two-photon FM) with Raman microscopy was published at around 2000 [13]. However, at that time, the timeframe for data acquisition for the analysis of a cell was a significant limitation of this technology, making the combined approach slower than the FM-based analyses. Accordingly, the system was inadequate for a high-throughput analysis of single-cells. Then, microscopy technology improved quickly, and most of the hardware limitations were eliminated, making the systems more stable [14]. Today, we can easily acquire high-resolution FM images to analyze the morphological and structural features of single cells, and at the same time, we can use Raman microscopy to obtain a chemical fingerprint of the same cells. Consequently, an in-depth analysis can be performed, and one can, for instance, reliably define mitotic events or the cellular state in general [15].

A single Raman spectrum might contain hundreds or even thousands of values, so analyzing a Raman map (which contains an array of such spectra) is a challenging task. Moreover, biological Raman data contain a plethora of peaks, most of them overlapping, which make the data even more complex and harder to work with. Therefore, to extract detailed information from the spectra, data analysis can benefit from machine learning approaches that are optimized for the analysis of high-dimensional data. Researchers have recently applied numerous different machine learning methods to preprocess and to analyze Raman spectra [16]. The most popular techniques are (*a*) principal components analysis (PCA), which is applied to simply reduce the dimensionality of the data, and (*b*) hierarchical component analysis (HCA), which is applied to find and to group similar spectra in the dataset. Furthermore, some newer techniques, such as t-distributed stochastic neighbor embedding (t-SNE) [17] are also applied to yield information from biological Raman data [18]. Other solutions were proposed in the literature [19]. However, the most promising methods for data analysis, as well as for preprocessing, are deep learning-based methodologies. Deep neural networks can denoise Raman spectra, resulting in a higher signal-to-noise ratio, thus shortening the data acquisition time and, consequently, rendering Raman spectroscopy a higher-throughput microscopy technique [20]. In addition, neural networks have the capability for speeding up the data analysis process and extracting useful information from hyperspectral data [21], making Raman spectroscopy a promising technique for clinical applications as well [22].

To critically understand the current limitations and opportunities related to correlative fluorescence and Raman microscopy (CFRM) when applied to define the biological statuses of the different cells composing a culture/tissue, we have performed several experiments in order to characterize the current weaknesses of a classical commercial system [23]. Via the correlative imaging of fixed cancer cells with stained nuclei, using a fluorescence microscope first and a Raman system afterwards, we have revealed that currently no validated procedures exist for (*a*) co-registering images between fluorescence and Raman microscopes; (*b*) normalizing the Raman spectra of different cells; and (*c*) identifying mitotic cells. Accordingly, here, we propose solutions for improved acquisition and analysis steps.

This study presents a comprehensive review of Raman spectroscopy in the field of cellular phenotyping, focusing on the correlative microscopy techniques and the applied AI-based data analysis methods. Furthermore, via a case study, the opportunities and the limitations of CFRM, combined with neural network-based data analysis, are examined. The following sections are organized as follows. Section 2 presents a short overview of earlier works using Raman spectroscopy for single-cell phenotyping. Section 3 describes cell classification methods based on correlative microscopy and machine learning approaches. In Section 4, we present a case study of a correlative system using deep learning approaches to analyze cell mitosis. Section 5 discusses the current opportunities and limitations related to CFRM. Finally, Section 6 reports on the main conclusions of this research work.

## 2. Raman Spectroscopy for Cellular Phenotyping 

Phenotyping has a fundamental role in biological research. Nowadays, several approaches are available to characterize a cellular system, even at the single-cell level [2]. An ideal technique should be non-destructive and it has the capability to reveal molecular changes within the sample. Practically, the intent of development is to extend the capabilities of microscopy techniques to yield more detailed information from a sample without compromising the cell status. Consequently, the question was raised as to whether Raman spectroscopy was applicable for this purpose. Based on the characteristics of this method, Raman microscopy is increasingly utilized to examine histological samples and cell cultures.

By acquiring Raman data, Matthäus et al. [24] analyzed the distribution of deoxyribonucleic acid (DNA) and proteins during cell division. Their goal was to monitor the distribution of chromatin and other biochemical components during mitotic events by recording molecular vibrations. They were the first researchers to report on the Raman and infrared (IR) micro-spectroscopic imaging of human mitotic cells. They imaged HeLa cells and applied both univariate and multivariate methods to visualize the distribution of cellular components. The deformation modes of uracil and thymine nucleic acids were employed to image the distribution of DNA, along with observing the spatial locations of proteins during mitosis. Furthermore, a hierarchical cluster analysis was performed using correlative Raman and IR data to make a hierarchical cluster map that captured the location of the nucleus within the cell. After the spectral data were recorded, nuclei were stained with DAPI to validate the results with FM. DNA-based Raman and DAPI-stained fluorescence images showed a high degree of correlation, indicating that Raman spectroscopy is an effective tool to map DNA distribution during mitosis without fluorescence staining. Although the results did not provide new insights into the process of mitosis, this study, along with other published ones, uniquely explored the whole cell division cycle by spectroscopic methods. However, the acquisition time for a single cell was 15 h, and this limitation prevented the utilization of this technology for the real-time high-throughput studies of live cells.

Later, Swain et al. [25] presented a pioneering method for the spectral characterization of biochemical changes in cell cycle dynamics at the single-cell level. They aimed to show that Raman spectroscopy is a proper tool to distinguish cells at different cell cycle stages. They used human osteosarcoma cells synchronized in the G0/G1, S, and G2/M phases. As a data analysis method, PCA was applied to identify spectral differences between cells in different phases. Then, using the significant principal components (PC) scores, a linear discriminant analysis (LDA) model was generated to classify the cells according to cell cycle phase. The PCA–LDA model showed a great spectral differentiation capability between the G0/G1 and G2/M phases. The model also demonstrated its ability for spectral discrimination between the G0/G1, and S or G2/M phases. Thus, Swain et al. have demonstrated the opportunities for the use of Raman micro-spectroscopy in their research of cell cycle dynamics, both for pharmacologically treated cells and in the case when the cell cycle is affected by cell–cell interactions. However, as these authors examined both the G0/G1 and G2/M phases in parallel, further research is needed to decide whether Raman spectroscopy can distinguish between the phases at this finer level of the cell cycle as well.

Similarly, Ichimura et al. [26] exploited the capabilities of Raman spectroscopy combined with PCA to examine cell state transitions during the cell differentiation process and to distinguish stem cells from differentiated ones. They investigated mouse embryonic stem cells (ESC) and compared the results with those for terminally differentiated cell lines, including fibroblasts, epithelial cells, and hepatocytes. Furthermore, they analyzed cells with a potential to differentiate, such as bone marrow mesenchymal stem cells (MSCs), adipocytes, and neuroblasts. They applied three approaches to examine these cells. On the one hand, they visualized the differences in Raman spectra that originated from the nucleus and the cytosol. In addition, they compared the averaged spectra of undifferentiated and differentiated cells, and they were able to distinguish them. On the other hand, they successfully discriminated between differentiated and undifferentiated cells by acquiring detailed Raman images for visualizing the presence of large lipid droplets and increased amounts of cytochrome C. Lastly, they visualized the PCA scores of the nuclear spectra of ESC, which were in a distinct state of differentiation. They could group the cells by their state, simply based on their PCA scores (PC1 and PC2). This indicates that Raman spectroscopy has the potential to distinguish cells in different phases of the cell differentiation process and to reveal molecular differences among them.

Finally, Pavillon et al. [27] presented an innovative method that enabled the non-invasive assessment of cellular changes in macrophages during their activation. They studied the activation process using a multimodal, label-free imaging approach which combines quantitative phase microscopy (QPM), Raman spectroscopy, and autofluorescence (AF) imaging. This way, they could provide quantitative information on both cellular morphology and molecular content at the single-cell level. Using their system, the cellular state or the extent of a response to a stimulus, such as lipopolysaccharide-induced macrophage activation could be measured, based on several label-free signals. Their experimental setup allows for the acquisition of QPM and AF images using an interferometric microscope, along with the recording of cellular Raman spectra. This approach was used to stimulate macrophages with lipopolysaccharide, and to generate statistical models based on the known states of stimulation from both morphological and molecular (Raman) data. The results for their applied models showed a fairly good correlation. This system was appropriate for identifying lipopolysaccharide-induced macrophage activation within a population of genetically identical cells. These findings indicate that Raman microscopy, compared with other classical morphology-based techniques (e.g., QPM or AF imaging), is a valuable tool for assessing actual cellular states such as the level of macrophage activation.

In conclusion, these works have demonstrated that spectroscopy techniques may have a broad area of applications to effectively improve in-depth biological research, ranging from distinguishing cell lines, through studying mitotic cells or examining cell differentiation, to exploring the molecular changes in biological processes.

## 3. Cell Classification with Correlative Microscopy and Machine Learning Approaches

Since Raman spectra and Raman images are high-dimensional data with a huge number of peaks from thousands of cell components, machine learning methods should have an essential role in processing and analyzing them. This holds true even more when the dataset is composed of information from different kinds of acquisition modalities. Merging Raman spectroscopy and other microscopy techniques can give new perspectives in biology and medicine, but the analysis of these datasets is not trivial, especially in the case of noisy and overlapping peaks. Moreover, it could be hard to apply Raman spectroscopy to certain sample types. Because of these obstacles, many authors focus on integrating new machine learning approaches into Raman data analysis, as well as on combining this approach with other types of microscopy techniques.

Harz et al. [28] presented an approach for single blood cell analysis in cerebrospinal fluid (CSF) samples, by applying both FM and Raman spectroscopy on the same sample. They aimed to figure out whether it was possible to characterize cells using FM in combination with Raman microscopy to gain additional molecular information. Using this technique, they first examined blood cells isolated from whole blood samples as a model system to determine several important methodological parameters. Next, they extracted immune cells from CSF and analyzed the influences of the CSF matrix on Raman spectra. Finally, they made a decision on the applicability of Raman spectroscopy for the analysis of CSF-derived blood cells. Their study demonstrated that Raman spectroscopy could be applied to fluorescence-stained blood cells, and they showed that the CSF matrix did not influence the final Raman interpretations. In addition, they successfully characterized single blood cells using PCA and HCA, and distinguished the DNA and protein-rich regions from the lipid-rich regions of the cell. This means that they could successfully discriminate spectra from the nucleus and the cytoplasm. However, the fluorescence of the applied dyes can mask the Raman signal of the cells. This could prevent the acquisition of information-rich Raman spectra. Therefore, choosing the appropriate dyes is fundamental.

Ahlawat et al. [29] analyzed the potential of Raman optical tweezers for label-free analyses of the cell cycle. They investigated whether an optical tweezer combined with Raman spectroscopy could be a proper tool for the examination of synchronized and fluorescence-stained mitotic cells. They merged Raman spectroscopy with fluorescence cytometry to monitor the molecular differences between cells in phases G0/G1 and G2/M. They found that by exploiting the intensity of some spectral bands, the DNA contents of the cells could be followed, and by using the Raman peaks of DNA, the different cell cycle phases could be distinguished. In addition, cells were differentiated according to their nuclear spectra using PCA. However, traces of the fluorescent stain were recognizable in the spectra; therefore, when using this technique, it is fundamental to exclude the peaks of the fluorescent dye from the analysis.

Other researchers [30] examined epigenetic changes in breast cancer by connecting Raman imaging, fluorescence imaging, atomic force microscopy (AFM), and scanning near-field optical microscopy (SNOM). Based on these findings, they discussed how the epigenetic changes contribute to different aspects of tumorigenesis in malignant and non-malignant human breast epithelial cell lines. They focused their attention on the information that can be extracted from cell nucleoli inside the nucleus and lipid droplets within the cell cytoplasm using Raman imaging. They analyzed the biochemical compositions of tumorous human breast epithelial cells and discussed the potential of Raman micro-spectroscopy as a tool to monitor epigenetic modifications in cancer development. The Raman spectra of nucleoli provide information about proteins: particularly, the vibrations of methyl and methylene groups in the region of 2800–3000 cm^−1^ are suitable to monitor the chromatin and non-histone type protein-related epigenetic modifications that are associated with different levels of acetylation or methylation.

On the other hand, Kallepitis et al. [31] developed a computational framework, namely qualitative volumetric Raman imaging (qVRI), which uses vertex component analysis (VCA) to execute the label-free visualization and quantification of cell components in 3D cell cultures. Using this approach, they could map and identify different lipid subtypes in monocytes and macrophages such as triglycerides, phospholipids and cholesterol esters. In addition, they could characterize the biochemical composition and morphology of the cells within a hydrogel biomaterial. Furthermore, the hydrogel matrix and the cell membrane could be distinguished by applying the framework and utilizing the capabilities of the system. They revealed that the bioactive hydrogel matrix had stronger interactions with the cells, as indicated by a more elongated cellular morphology in 3D, while such a finding was not evident for the bioinert hydrogel material. In summary, they showed that Raman spectroscopy combined with VCA has the capability to reveal valuable information about cell differentiation or potentially any other biological processes, even in a 3D manner.

Another approach tested by Krauss et al. [32] builds on applying deep convolutional neural networks (DCNN) to Raman microscopic image analysis. They analyzed the Raman spectra of cancerous and non-cancerous urothelial cells from the sediments of urine samples to decide whether neural networks could serve as an appropriate classifier of spectral images, and whether image spectra can distinguish cancer cells from healthy ones. They used *AlexNet* topology [33] as a starting point and derived three additional variants of this network. Using these DCNNs and some selected wavenumbers from the data, the urine samples were classified as cancerous or healthy ones. As a comparison, two conventional classifiers were applied to the same dataset in order to serve as a baseline approach. In contrast to the original *AlexNet* topology which yielded poorer results than conventional classifiers, the derived neural networks outperformed the accuracies of conventional techniques. Considering these findings, neural networks could serve as the classifiers of choice to interpret spectral data. However, acquiring a huge amount of data, which is essential for neural networks to achieve high accuracy, not only at some selected wavenumbers, but in the whole spectra, is a time-consuming process because of the weak Raman signal. Therefore, using neural networks for Raman spectral images has not become a widespread technique.

Kobayashi-Kirschvink et al. [34] presented a unique experimental and computational framework which can predict the expression profiles of single live cells using Raman microscopy. These machine learning models were trained to infer gene expression levels from Raman spectra. For this purpose, the researchers used ribonucleic acid–fluorescence in situ hybridization (RNA-FISH) data as anchors to link single-cell RNA sequences to paired Raman spectra. As a machine learning method, they used their own deep learning approach developed previously [35] to link spatially resolved FISH (smFISH) data to RNA sequences, after applying *Catboost* [36] to deduce anchor expression levels from the Raman data. Their framework was appropriate to predict expression profiles with high accuracy from Raman hyperspectral data, both at the cell type and at the single-cell levels.

## 4. Raman Imaging of Mitosis: A Case Study

**Figure 1:**
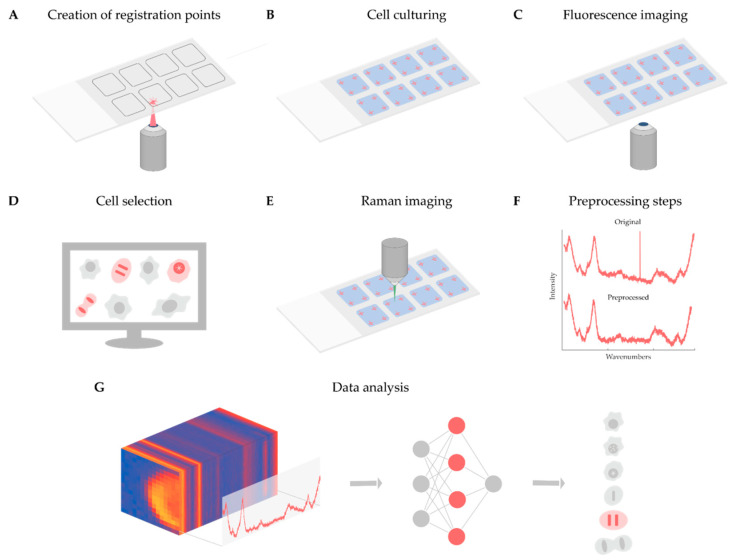
(**A**) Registration points were designated using a laser microdissection microscope. (**B**) Cell culturing was executed in plates ready for imaging, with 4 registration points in each chamber. (**C**) Fluorescence images were acquired with a confocal microscope. (**D**) Cell selection was executed by an expert. (**E**) Acquiring data with a Raman microscope. (**F**) Preprocessing Raman data, including cosmic ray removal, standardization, and fitting. (**G**) Neural network training for supervised classification of mitotic stages.

As discussed in detail above, Raman-based imaging is suitable for acquiring data on spatially distributed chemical characteristics, giving a complex set of information about the compounds comprising the material under examination. Fluorescence image analysis is an established method to quantify cells, although it does not give direct molecular information about the sample, and most of the time, fluorescent dyes are toxic to the examined biological system when applied to live cells. In contrast, Raman microscopy can give detailed molecular information in a non-invasive way. For instance, it is appropriate to analyze the DNA content of single cells and to define the biological status of the cell of interest. However, the Raman spectra obtained are noisy and require normalization, as well as other data preprocessing steps, e.g., cosmic ray removal, standardization, and spectrum fitting. To better understand the current limitations and opportunities of the technique, we present a case study on the analysis of Raman maps. The main aim of the project was twofold. Firstly, determining whether a cell is in the interphase or the mitotic phase. Then, for mitotic cells, extracting the exact mitotic phase (pro-, prometa-, meta-, ana-, and telophase). In our experiment, we solved these tasks as a classification problem with six classes (one for the interphase and five others for the mitotic phases). The classifier was implemented in the form of a two-dimensional (2D) convolutional neural network. Currently, fluorescence images are used for classifying the cells, to create a ground truth. In the future, we will use label-free screening to detect the cells of interest. Below, we present a summary of the experiments performed, schematized in Figure 1.

### 4.1. Experimental Setup

***Cell Culturing and Nuclei Staining***: For the case study, Henrietta Lacks (HeLa) cells (Chemokines C–C motif chemokine ligand 2, CCL-2, the cell line derived from American Type Culture Collection, ATCC) were cultured on glass slides using 8-well FlexiPerm silicon chambers. HeLa cells were grown in Eagle’s minimal essential medium (EMEM) supplemented with 10% fetal bovine serum (FBS). Around 15,000 cells were seeded into each chamber and incubated at 37 °C in a 5.0% CO_2_ humidified environment for 24 h. After washing with phosphate-buffered saline (PBS, pH7), the cells were fixed with 4% paraformaldehyde (PFA) at room temperature, dissolved in PBS for 10 min, and washed with PBS 3 times for 2 min. After washing, the nuclei of the cells were stained with DAPI (1µg/mL) for 30 min.

***Fluorescence Imaging*:** Widefield fluorescence and brightfield images were obtained using a Leica TCS SP8 confocal laser scanning microscope equipped with a 20× air objective (numerical aperture, NA: 0.4; working distance 6.9 mm). Images were acquired with a 1.4 MP monochrome charge-coupled device (CCD) digital camera (Leica, DFC365 FX). For detecting nuclei staining, DAPI excitation and emission filters were used (i.e., 350/50 and 460/50, respectively). The screening was performed with a 10% overlap of the images and a laser-based autofocus system.

***Identifying Mitotic Cells:*** First of all, mitotic cells were manually identified by an expert microscopist under the fluorescence microscope. To identify the same cells with the Raman microscope, four registration points were designated in each well of the multi-well plate before the cells were plated on the glass slide. The registration points were engraved into the glass support with a laser of a Zeiss photo-activated localization microscopy (PALM) MicroBeam microscope with a 40×/0.6 NA objective (Zeiss, Germany). Then a projective transformation was estimated for each well using our software, and the transformation of the coordinates between the microscopes was performed. The transformation was executed by our custom-built software. Then, using the transformed positions, the cells selected under the brightfield microscope were detected again with the Raman microscope. 

***Raman Imaging***: Raman imaging was performed on an NT-MDT Spectrum Instruments Ntegra II Raman–AFM microscope system (NT-MDT, Russia) equipped with a 60×/1.0 NA water immersion objective (Olympus, Japan). The light source was a 532 nm laser with a power of 12 mW above the objective. The scattered light was detected with a thermoelectrically cooled CCD at –70 ℃ (Andor, UK) via 300 grooves per mm diffraction grating. The spectra were acquired within the range of 439 cm^−1^ to 3228 cm^−1^. The acquisition time of the Raman spectra was 1.2 s, with a spatial resolution of 1 µm. Each spectral map spanned 24 µm × 24 µm and was cropped to remove the neighboring cells and most of the background.

**Figure 2:**
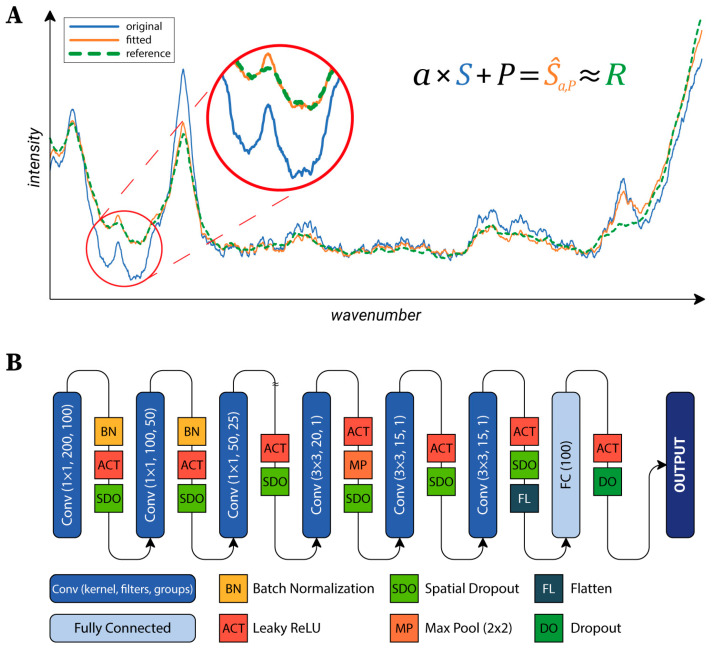
(**A**) Visual demonstration of the proposed fitting method. The magnified part highlights a section where significant improvement is visible. (**B**) The architecture of the utilized neural network. The first 3 layers reduce the number of channels with 1×1 kernels, while the next layers apply “classic” convolution with a 3×3 kernel size.

***Preprocessing****:* After data acquisition, preprocessing was performed. First, spectral maps were revised to remove the measurements related to the regions not containing cells (because of washing issues), or to regions with fluorescent signals strong enough to hide the Raman spectra of the cells. Furthermore, the spectral maps were cropped to cover a single cell for each map. Second, most of the cosmic rays were removed from the data using an algorithm proposed by Cappel et al. [37]. From a preliminary experiment on a subset of data, we verified that it was able to correctly remove approximately 95% of the cosmic rays (an expert operator manually highlighted all of the cosmic rays to create a ground truth for the efficiency evaluation). Accordingly, we decided to adopt this algorithm for cosmic ray removal. This step was complemented with manual removal of the remaining cosmic rays based on visual inspection to eliminate the cosmic rays which were not detected by the algorithm. The next step was to normalize each spectrum to a similar intensity. Two different intensity correction methods were tested, standard normal variate (SNV) [38] and baseline correction using polynomial fitting [39], but none of them produced acceptable results for the data analyzed. A quantitative analysis with representative examples can be found in Section 4.1. Thus, we propose an original algorithm (the implementation is freely available at *https://github.com/biomag-lab/raman-fitting*), which fits each spectrum to the same reference. This was an arbitrarily selected background spectrum, but the average of the background spectra might have also been used. The transformation of a spectrum is given in the following form (Equation (1)):

(1) $${\hat{S}}_{a,P}=a\times S+P,\;\;\;\;\;\;\;P\left(j\right)=p\left(x_{j}\right),\;\;j=1,\ldots ,m,\;\;p\left(x\right)=\overset{n}{\underset{i=0}{\sum }}p_{i}x^{i}$$

where *S,Ŝ∈**ℝ^m^*** are the original and the transformed spectra, respectively, *P∈**ℝ^m^*** is a sampling of the polynomial *p(x)*. *m* is the number of values in the Raman spectra; *n* is the order of the polynomial. In our experiments, we found that the algorithm worked best on our data, with *n = 5.* The parameters of the transformation (*ɑ∈**ℝ*** and the *p_i_∈**ℝ**, i=0,…,n* coefficients) are found by solving the optimization problem (Equation (2)):

(2) $$\underset{a,P}{\mathrm{argmin}}\frac{1}{2}\overset{n}{\underset{i=1}{\sum }}L_{\delta }\left({\hat{S}}_{a,P}\left(i\right)-R\left(i\right)\right)\;L_{\delta }\left(x\right)=\left\{\begin{array}{cc} x^{2}, & \;\;\left|x\right|\leq \delta ,\\ 2\delta \left|x\right|-\delta^{2}, & \;\;otherwise \end{array}\right.$$

for a previously defined reference spectrum *R* (see Figure 2A). The *L_δ_* function is the Huber loss [40]. This loss function is a combination of the mean squared error (MSE) and the mean absolute error (MAE). The main advantage of this loss function is that it is insensitive to outliers, in contrast with MSE, which would highly penalize greater errors. In our case, this means that the significant peaks were ignored during fitting, and only the baseline was matched to the reference spectrum. To provide a generally applicable *δ* value, the SNV transform was applied to each spectrum. After that, *δ* = 0.02 was used. The quality of the proposed fitting method was evaluated as described in Section 4.2.

***Neural Network Architecture***: To classify the cells, a neural network (NN) was designed. Raman maps can be considered as images with 1600 channels (the number of values per a single spectrum). Accordingly, the main structure of the utilized NN’s architecture is similar to the general networks used in the field of image processing (see Figure 2B) [41]. The first part of the network consists of convolutional layers, each reducing the dimensionality of the data, which are connected to the second part composed of two fully connected layers, which produce the output. The first part can be further divided into two sections. The first section hierarchically reduces the number of channels by grouping convolutional layers with a kernel size of 1×1. The second section is composed of convolutional layers which do not use grouping and that work with a kernel size of 3×3. Data were fed into the network in 15×15 crops, which were created during the training to reduce memory consumption. Cropped regions were shifted from each other by two pixels, thus, different parts of the same cell were utilized as training data (test and validation data were left intact).

### 4.2. Results

**Figure 3:**
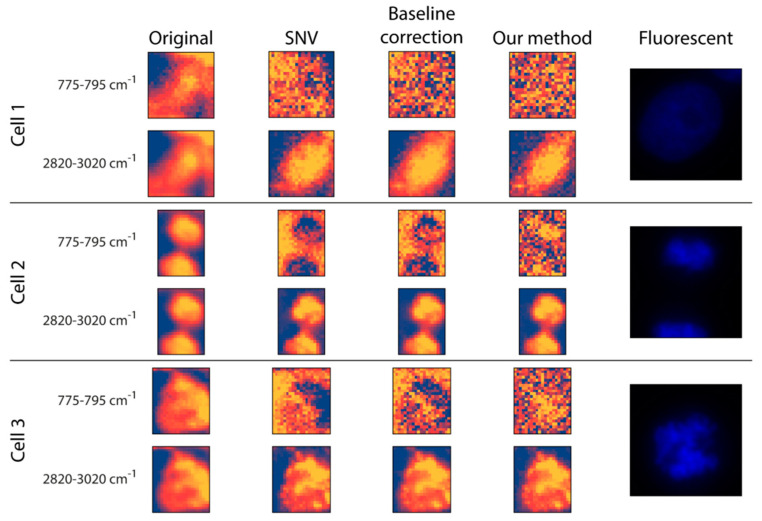
Comparison of the preprocessing methods tested. The same wavenumber intervals were used for the cells: 775–795 cm^−1^ for DNA, and 2820–3020 cm^−1^ for proteins and lipids. Each cell is in a different mitotic phase (inter-, telo-, and prometaphase, respectively). The last column shows the fluorescence (DAPI) image for reference.

***Preprocessing:*** The original data and two commonly used Raman preprocessing methods (i.e., SNV and baseline correction) were compared with the proposed fitting method (Figure 3). For inspection, two wavenumber intervals were displayed: (*I*) 775–795 cm^−1^ for the nucleus and (*II*) 2820–3020 cm^−1^ for (mostly) lipids and proteins. (*I*) is dominated by the pyrimidine ring breathing, which is a characteristic mode of vibrations of these DNA compounds, whilst (*II*) is dominated by the vibration modes of CH_2_ and CH_3_ groups which are overrepresented in lipids [42,43,44]. Regarding the raw data, there is no substantial morphological difference between the two intervals. Additionally, it is worth mentioning that none of them had any texture as such, but the cells appeared to be quite homogeneous. This was improved at the 2820–3020 cm^−1^ wavenumber interval by all three preprocessing methods, as they seemed to introduce some textural information for the cytoplasm. This was not the case for the nucleus interval, where the three preprocessing methods yielded quite different results. For *Cell 1*, none of the preprocessing methods was able to highlight the nucleus, probably because the DNA was not concentrated in a smaller, dense area. For *Cells 2* and *3,* the SNV method made a larger proportion of the cell appear at a lower intensity compared to the background, with no clear sign of the nucleus. Similar to SNV, the baseline correction made some of the cell pixels less intense compared to the background positions; however, here we obtained a glimpse of what appeared to be the nucleus, meaning that it was capable of some sort of a separation between the nucleus and the rest of the cell. Thus, although it cannot be regarded as being of high quality, the proposed fitting method was able to differentiate the nucleus from both the background and other cellular compartments.

**Figure 4:**
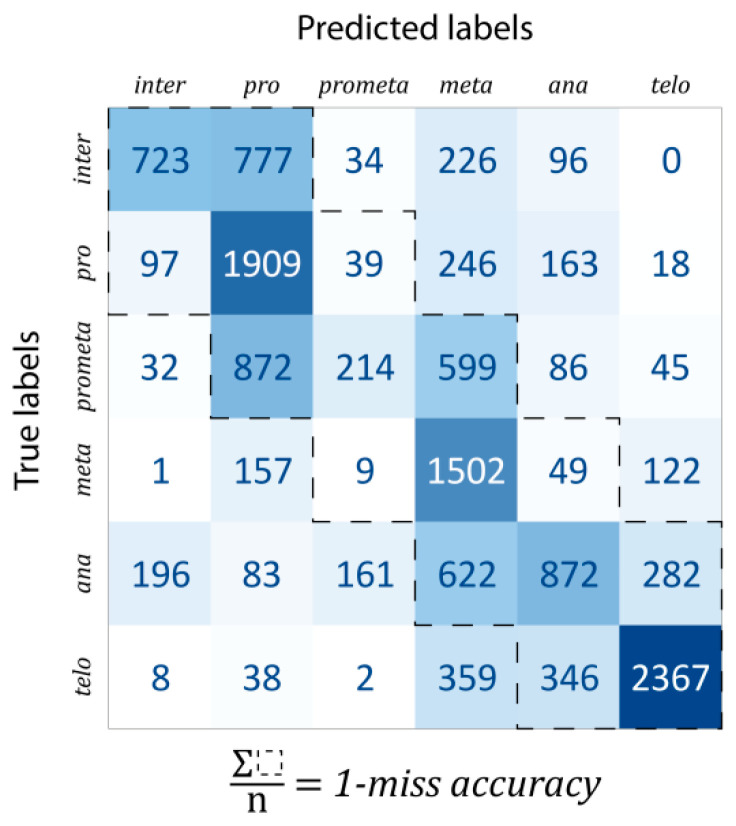
Confusion matrix of the trained model.

***Classification***: For evaluation, the data were split into train–test–validation sets in a 70%–15%–15% ratio. The confusion matrix of predictions is shown in Figure 4. The one-miss accuracy was 84.47% (i.e., exact hit or hitting the neighboring mitotic stage), while the standard accuracy of the trained model was 56.82%. There are some remarks which should be noted. Firstly, some classes are much harder to learn than others. For example, prometaphase was mostly classified into either pro- or metaphase (47.19% and 32.41% of all prometaphase cells, respectively, while the proportion of correctly classified cells was only 11.58%). This is likely explained by the fact that prometaphase (as the name suggests) is an intermediate stage between the pro- and the metaphases. In general, most of the misclassifications happened for consecutive phases. For this reason, a better characterization of the predictive capabilities of our model, such misclassifications were considered by calculating the one-miss accuracy value, for which the sum of the tridiagonal elements of the confusion matrix was divided by the total number of data units.

## 5. Discussion: Opportunities and Limitations

In this work, we have focused on the detection of mitotic stages at the single-cell level using CFRM and artificial intelligence approaches. In particular, we have analyzed state-of-the-art works and case studies to understand the current limitations and opportunities of the field. Our findings are summarized below.

***Fluorescence Imaging:*** Fluorescence microscopy is a mature and widely used technique in the field of biology. Its simplicity and high-throughput nature make this technique an almost perfect tool to gather information from a biological system. However, it has some limitations. One of the drawbacks of fluorescence microscopy is the potential for the overlapping spectra of the applied dyes, therefore, the simultaneous investigation of multiple target molecules can be difficult or even impossible. In addition, fluorescent tags can interfere with molecular transport in live cells; thus, the observation can lead to false conclusions. Moreover, the applied dyes can be cytotoxic and may damage the specimen.

***Raman Imaging:*** One of the advantages of Raman spectroscopy is that it does not require staining and it allows for the parallel assessment of multiple target molecules. Accordingly, live cell molecular imaging can be performed without the disturbing effect of staining. On the other hand, the Raman signal is typically noisy. Therefore, it needs a longer integration time to improve the signal-to-noise ratio. In addition, the biological samples are in general molecularly heterogeneous, causing the peaks in spectra to overlap. These properties make the biological interpretation of Raman signals a challenging task.

***CFRM:*** CFRM is a useful tool in molecular biology. By combining fluorescence images with spectral data, the morphological and molecular features of the sample can be inspected together to obtain more comprehensive knowledge about the investigated sample. On the other hand, combining multiple imaging tools introduces difficulties, as multiple hardware/software elements need to be connected. Image registration is needed because the same cells are measured with each modality, which can be hard due to the complexity of Raman data. After solving this issue, information-rich data are at hand for further analysis.

***Cell Selection:*** Choosing the right cells for measurement is a crucial question. Manually selecting areas to measure is tedious work. This can be solved by using automated image analysis, and AI-based methods may offer a favorable solution to optimize the data acquisition process and select the representative cells for the assessment. Currently, there is no generally accepted machine learning procedure, but several solutions exist [28,31,45]. For instance, the commercial software called *BIAS* (Single Cell Technologies L.T.D., Szeged, Hungary) provides an AI model to predict the mitotic phases of dividing cells, and an interface to connect CFRM devices. As proof of concept, we have collected and annotated fluorescence images to guide Raman data acquisition. We have built a database containing more than 6000 fluorescence images and 2000 annotated mitotic cells. Then, we analyzed images using *BIAS* to predict the mitotic phase (85% recognition accuracy and 10-fold cross-validation) and to guide the Raman microscope. Consequently, AI offers a real opportunity to make Raman imaging faster by assessing only the cells of interest.

***Microscope Registration:*** Registering multiple microscope systems is required whenever one aims to combine information from multiple modalities. Various techniques differ in flexibility and complexity; however, as the models become more complex (to be able to handle difficult transformations), their degree of freedom rises, making optimization difficult. Upon moving a sample between microscopes, the simplest way to avoid distortions (such as bending, stretching, drying of the sample, etc.) is through using marker points to find the corresponding positions. The type of the required transformation (rigid, affine, and projective) depends on the setup (unit of measure, relative positions of the sample and the objective, etc.). The main sources of registration error come from the mislocation of registration points (due to user error, or because the marker points are not negligibly thin) or the hardware limitations of the motors moving the sample. While these errors can cause problems in the downstream steps of the pipeline, these rather small mistakes can be corrected, either manually or via a (semi)automatic method.

***Data Preprocessing:*** Raman spectroscopy is subject to several types of distortions, which might compromise the model’s accuracy; thus, those should be eliminated. First of all, cosmic rays are particles coming from outer space with high energy. As they hit the atmosphere, multiple particles emerge, which can cause huge energy spikes when hitting the CCD of the spectroscope, but numerous techniques are available to remove these spikes from the spectra [46,47,48]. Noise is another problematic issue. As discussed above, the signal-to-noise ratio can be improved by increasing the acquisition time. While this is a simple solution, it negatively impacts the amount of data that one can gather in a reasonable timeframe. Therefore, deciding on an appropriate acquisition time is a compromise between data quantity and quality. That is why assessments often produce noisy spectra, making data analysis more difficult. There are several solutions to reduce noise [49], but care must be taken, as radical noise reduction might remove useful information as well. It is worth mentioning that more advanced smoothing techniques also exist, which might utilize neural networks [50]. Unfortunately, other types of destructive impacts are not so straightforward to handle. Fluorescence adds a baseline to the overall spectrum, drastically modifying the shape of the spectra, which hinders comparison studies. In addition, the power of the laser used is subject to fluctuation, causing the overall intensity of the spectrum to drop or increase unpredictably. There are a plethora of tools to tackle these challenges, but it is hard to choose the best option. Simple techniques, such as SNV, are easy to apply to the data at hand. The lack of parameters is both a blessing and a curse, in that the output is produced consistently, but it lacks the flexibility to remove serious distortions such as fluorescence. Some of the available baseline removal algorithms use polynomials to define the shape of the background signal [51,52,53], thereby introducing a parameter that is difficult to optimize. When a polynomial order is too low, it is inappropriate to represent the baseline precisely, but setting it too high will overfit the spectrum, introducing wave-like artifacts upon subtraction. Using a reference spectrum for fitting may also be a reasonable choice, but it comes with the risk of forcing spectra to be “too similar” to the reference, discarding the existing information from the data.

***Data Analysis:*** Raman maps can be considered digital images with hundreds, or even thousands of channels, making it a complex type of data due to its high dimensionality. Therefore, applying machine learning techniques can improve data analysis. For instance, deep-learning methods can recognize complex patterns within the data, and can reveal hidden relationships between data points; thus, they support a more efficient data analysis. This is in contrast with general AI techniques, where predefined features need to be extracted from the raw data. The introduced technique can analyze whole Raman spectral maps containing hundreds of thousands of numeric values. By eliminating the need for handcrafted features, the NN is able to extract information from the data, which could remain undetected during a manual inspection. In addition, the architecture can investigate spectral and spatial features together, allowing a more thorough analysis. Another issue to consider is that, although originally NNs are not human-interpretable (it is hard to check what features were used for the decisions), there are techniques to visualize areas in images, which obtained a bigger emphasis from the neural networks (e.g., Grad-CAM [54]). This can help in understanding biological processes at a molecular level. One downside of deep NNs is that they are “data hungry” which can limit their application because gathering huge amounts of Raman data is time-consuming. However, when a sufficient amount of data are available, combined with the appropriate data preprocessing and architecture, NNs appears as an effective tool in advanced data analysis. Accordingly, developing machine learning techniques is a hot topic nowadays, and several research groups are engaged in developing new ways to expand the capabilities of these techniques, or custom solutions for spectral analysis.

## 6. Conclusions

Single-cell level morphology and molecular analyses play a significant role in the advancement of biology and medical research. For instance, current oncological drug development is based on studying the effects of chemical compounds on cells of different phenotypes, and on cells in different cellular stages. Microscopy techniques, which enable biologists to analyze cellular and subcellular components, have provided valuable information for many years, but currently, no single microscope is capable of analyzing all aspects of the cells in their complex environment.

For a comprehensive, in-depth analysis of a sample, a combination of advanced instruments is required. The location and morphology of stained molecules can be obtained using fluorescence microscopy. Label-free images, informing on the spatial distribution of molecular fingerprints at the subcellular level, can be produced with Raman microscopy. CFRM, the combination of these two techniques, offers a unique opportunity for the in-depth assessment of cellular stages at the single-cell level. In addition, artificial intelligence can speed up the process by selecting representative cells for the analysis. Nevertheless, several limitations still leave the practical and reliable applications of CFRM an open challenge.

In order to understand how successful research has merged fluorescence microscopy, Raman spectroscopy, and artificial intelligence for cellular phenotyping, we have reviewed state-of-the-art articles published on this topic. Then, we executed a case study to reveal the restrictions limiting the application of CFRM to classify mitotic cellular stages based on the spectral map. Despite the identified difficulties, promising results were presented. We hypothesize that by applying more advanced AI-based techniques, such as transfer or semi-supervised learning, better results can be achieved in the future.

Based on our findings, we can conclude that currently, there is no generally accepted procedure for (*a*) co-registering images between fluorescence and Raman microscopes; (*b*) normalizing the Raman spectra of different cells; and (*c*) identifying mitotic cells. On the other hand, numerous solutions are appearing for all these steps, creating high expectations for a truthful application of CFRM in the near future.

## Data Availability

The data that support the findings of this study are available from the corresponding author upon reasonable request.

## Abbreviations

American Type Culture Collection	ATCC
artificial intelligence	AI
atomic force microscopy	AFM
autofluorescence	AF
cerebrospinal fluid	CSF
charge-coupled device	CCD
chemokines C–C motif chemokine ligand 2	CCL-2
correlative fluorescence and Raman microscopy	CFRM
deep convolutional neural networks	DCNN
deoxyribonucleic acid	DNA
Eagle’s minimal essential medium	EMEM
electron microscopy	EM
embryonic stem cell	ESC
fetal bovine serum	FBS
fluorescence in situ hybridization	FISH
fluorescence microscopy	FM
Henrietta Lacks	HeLa
hierarchical component analysis	HCA
infrared	IR
light microscopy	LM
linear discriminant analysis	LDA
mean absolute error	MAE
mean squared error	MSE
mesenchymal stem cell	MSC
neural network	NN
numerical aperture	NA
phosphate-buffered saline	PBS
photo-activated localization microscopy	PALM
principal components	PC
principal components analysis	PCA
qualitative volumetric Raman imaging	qVRI
quantitative phase microscopy	QPM
ribonucleic acid	RNA
scanning near-field optical microscopy	SNOM
spatially resolved FISH	smFISH
standard normal variate	SNV
t-distributed stochastic neighbor embedding	t-SNE
two-dimensional	2D
vertex component analysis	VCA
